# Role of aerobic physical training on cardiac autonomic and morphophysiological dysfunction in hypertensive rats subjected to ovarian hormone deprivation

**DOI:** 10.1590/1414-431X2022e11916

**Published:** 2022-05-16

**Authors:** B.R.O. Rossi, S.V. Philbois, K.D. Maida, J.C. Sánchez-Delgado, A.C. Veiga, H.C.D. Souza

**Affiliations:** 1 Laboratório de Fisiologia do Exercício e Fisioterapia Cardiovascular, Departamento de Ciência da Saúde, Faculdade de Medicina de Ribeirão Preto, Universidade de São Paulo, Ribeirão Preto, SP, Brasil; 2 Faculty of Physical Culture, Sports and Recreation, University Santo Tomás, Bucaramanga, Colombia

**Keywords:** Cardiovascular autonomic control, Echocardiography, Hypertension, Menopause, Physical training

## Abstract

Here we investigated the effects of physical training on cardiovascular autonomic control and cardiac morphofunctional parameters in spontaneously hypertensive rats (SHRs) subjected to ovarian hormone deprivation. Forty-eight 10-week-old SHRs were divided into two groups: ovariectomized (OVX, n=24) and sham (SHAM, n=24). Half of each group (n=12) was trained by swimming for 12 weeks (OVX-T and SHAM-T). Cardiac morphology and functionality were assessed using echocardiography, and autonomic parameters were assessed using double pharmacological autonomic block, baroreflex sensitivity (BRS), and analyses of heart rate variability (HRV) and blood pressure variability (BPV). Ovariectomy did not influence the cardiac autonomic tonus balance unlike physical training, which favored greater participation of the vagal autonomic tonus. Ovariectomy and aerobic physical training did not modify HRV and BRS, unlike BPV, for which both methods reduced low-frequency oscillations, suggesting a reduction in sympathetic vascular modulation. Untrained ovariectomized animals showed a reduced relative wall thickness (RWT) and increased diastolic and systolic volumes and left ventricular diameters, resulting in increased stroke volume. Trained ovariectomized animals presented reduced posterior wall thickness and RWT as well as increased final diastolic diameter, left ventricular mass, and stroke volume. Ovarian hormone deprivation in SHRs promoted morphofunctional adaptations but did not alter the evaluation of cardiac autonomic parameters. In turn, aerobic physical training contributed to a more favorable cardiac autonomic balance to the vagal autonomic component and promoted morphological adaptations but had little effect on cardiac functionality.

## Introduction

Cardiovascular diseases (CVDs) have high morbidity and mortality rates and are considered a major public health problem worldwide that affects both men and women (1). Although CVDs share several modifiable risk factors, such as obesity, smoking, physical inactivity, and eating habits, women of reproductive age are less likely to develop these diseases than men and postmenopausal women (2). This suggests that ovarian hormones, especially estrogens, play an important cardiovascular protective role (3-5). Although ovarian hormonal protection prevails at reproductive age, some women develop CVDs such as systemic arterial hypertension (SAH) that can determine a series of cardiovascular impairments that may compromise cardiovascular homeostasis.

Among these impairments, adverse cardiac morphological and functional adaptations and cardiovascular autonomic control changes are the most common and often associated with a less favorable prognosis (3-6). However, little is known about the contribution of ovarian hormones to autonomic and morphofunctional cardiac protection due to SAH. This statement is based on experimental studies that show the importance of ovarian hormones in the regulation of the sympathetic autonomic drive and renin-angiotensin-aldosterone system activity in addition to their important role in endothelial function, fibrosis control, and adverse cardiac hypertrophy (5,7,8).

On the other hand, regular physical exercise is a non-pharmacological tool in the prevention and control of chronic degenerative diseases including SAH (7,9,10). Specifically, the effects on the cardiovascular system of exercise, especially aerobic physical training, include a number of benefits such as reduced blood pressure, improved autonomic tonic balance over the heart, and beneficial cardiac morphological and functional adaptations in addition to the increase in myocardial angiogenesis and optimization of mitochondrial density and endothelial function (7-9,11,12).

In this sense, this study aimed to investigate the therapeutic role of aerobic physical training in cardiovascular autonomic control, cardiac morphology, and functionality in spontaneously hypertensive rats (SHRs) subjected to ovarian hormone deprivation.

## Material and Methods

### Animals

**Figure 1:**
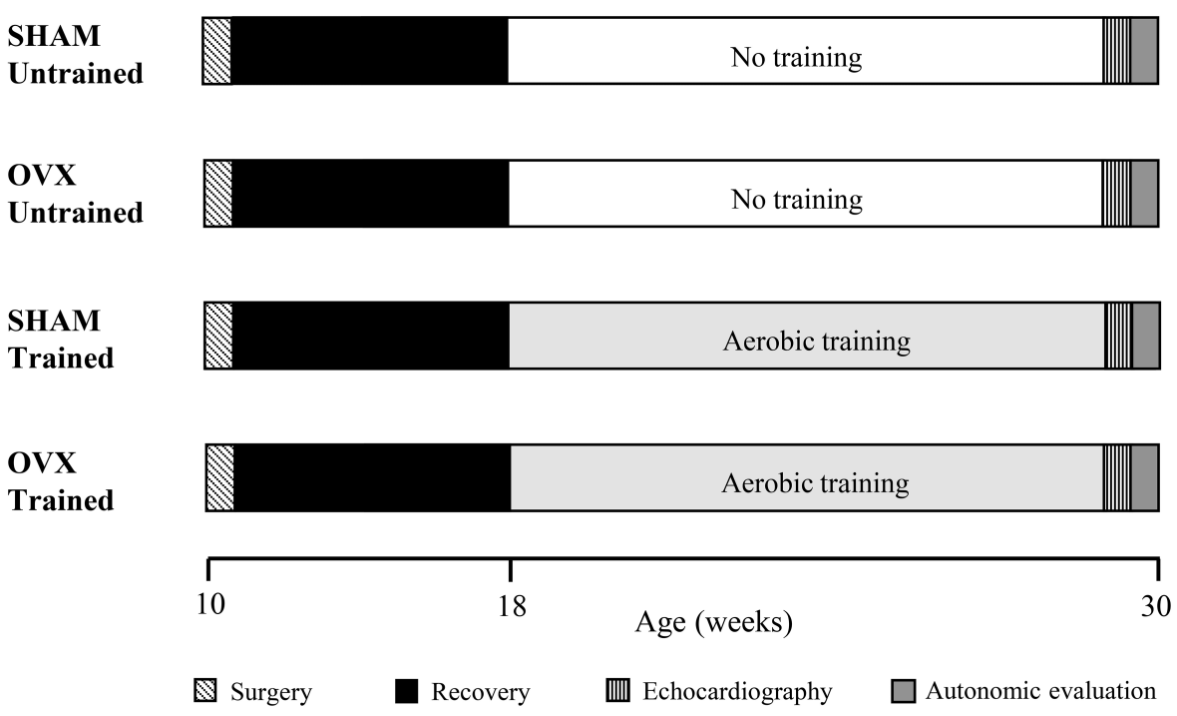
Schematic representation of the experimental timeline of SHAM and ovariectomized (OVX) groups.

Ten-week-old female SHRs (n=48) weighing 150±5 g obtained from the Central Vivarium of the University of São Paulo (USP), Ribeirão Preto Campus, were used. The animals were divided into ovariectomized (OVX, n=24) and SHAM surgery (SHAM, n=24) groups. Half of each group (n=12) was subjected to aerobic physical training for 12 weeks through swimming (OVX-T and SHAM-T) (Figure 1). The initial age of the animals was based on the start of puberty, marked by the vaginal opening followed by the first ovulation and the subsequent ovulatory cycle, a process that occurs at approximately 50-60 days of life (13-15).

During the experimental period, the animals were housed in the vivarium of the Postgraduate Program in Rehabilitation and Functional Performance with a 12-h light/dark cycle, temperature of 21±1°C, and free access to water and food (Nuvilab CR-1, Nuvital, Brazil).

The experimental protocols used in the present study were performed in accordance with the ethical principles of animal experimentation adopted by the Brazilian College of Animal Experimentation and evaluated and approved by the Animal Experimentation Ethics Committee of the Ribeirão Preto Medical School (Brazil) (protocol 148/2019).

### Surgical procedure

At 10 weeks of age, the rats were anesthetized with 80 mg/kg ketamine and 10 mg/kg xylazine, and a small abdominal incision was made. The ovaries were located, and a silk thread was tightly tied around the oviduct to include the ovarian blood vessels.

The oviduct was sectioned, and the ovaries were removed. The contralateral ovary was removed in a similar manner. The sham surgery rats underwent the same procedure except for the sectioning of the oviducts and removal of the ovaries. The skin and muscle walls were then sutured using silk thread. All animals received prophylactic antibiotic therapy (40,000 IU, intramuscular; Vitalfarma, Brazil) following the surgical procedures. The rats were housed individually, and an 8-week postoperative recovery period was allowed. After recovering, the rats were housed in groups of three per cage (60×50×22 cm, Insight Ltda., Brazil). Daily vaginal smears were collected from all rats as previously described (16). This procedure allows for the determination of the estrous cycle phase by the daily analysis of the cells that slough off the vaginal epithelium at four different stages: proestrus (nucleated epithelial cells), estrus (cornified cells), metestrus (some cornified cells and other nucleated cells accompanied by a large number of leukocytes), and diestrus (leukocyte infiltration). The collected vaginal fluid was placed on glass slides and examined under light microscopy (40× magnification). The absence of the estrous cycle in the ovariectomized groups was confirmed by the presence of a permanent diestrus phase.

### Physical training

Training occurred between 7:00 and 10:00 am. The trained groups underwent an aerobic physical training protocol that consisted of swimming sessions in a glass tank (100×80×80 cm) that enabled the simultaneous training of six animals. The tank was filled with 50 cm of warm water (30±2°C), which was changed after every group training session. The training program was conducted in two different stages over a total of 12 weeks. The first stage consisted of a 2-week adaptation period, during which the session length was gradually increased from 5 to 45 min (5 min per day) five times per week. The second stage consisted of 10 weeks of 45 min of physical training five times per week. To evaluate training intensity, blood was collected from the tail vein of the animals at weeks 3, 5, and 10 immediately before and 15 min after the exercise, and lactate concentrations were measured (Accutrend^®^ Plus, Roche Diagnostics, Germany). The expected lactate level was 5.5-6 mM, as described previously (17,18). If the animals did not reach the expected lactate concentration, training exertion was increased by the fastening of an impermeable lead-containing Velcro strap to the chest to increase body weight by 2-6% (17).

### Experimental protocols

#### Echocardiography

All animals were subjected to an echocardiographic evaluation at 30 weeks of age. We used a Vevo 2100^®^ High-Resolution Imaging System (VisualSonics, Canada) with a high-resolution transducer (21 MHz). For the procedure, the anterior regions of the thorax were previously trichotomized (Veet^®^, Reckitt Benckiser, Brazil) and all the animals were anesthetized with 1.5% isoflurane supplemented with 1% O_2_ and placed on a heated (37°C) platform. Echocardiography and temperature measurements were also performed.

High-resolution B-mode and M-mode images were acquired. Wall thickness and left ventricle dimensions were obtained on short-axis view at the level of the papillary muscles. Diastolic measurements were performed at the point of maximum cavity dimension, and systolic measurements were performed at the point of minimal cavity dimension. All measurements were performed according to the American Society of Echocardiography standards (19) by an evaluator who was blinded to the group assignments. The following parameters were obtained from the images: interventricular septum thickness (IVST), posterior wall thickness (PWT), left ventricular end-diastolic diameter (LVEDD), and left ventricular end-systolic diameter (LVESD). The shortening fraction was calculated as follows: SF (%) = [(LVEDD − LVESD ÷ LVEDD) × 100], and the ejection fraction (EF) was calculated using the Teichholz method: [(LVEDV - LVESV ÷ LVEDV) × 100] (20). The left ventricular mass (LV mass / final body weight) was calculated as 1.047 × [(LVEDD + PWT + IVST)^3^ − (LVEDD)^3^] (21), while the relative wall thickness (RWT) was calculated as [2 × PWT ÷ LVEDD]. Left ventricular volumes were quantified as LVEDV (μL) = [LVEDD^3^ × (7 ÷ 2.4 + LVEDD^3^)] and LVESV (μL) = [LVESD^3^ × (7 ÷ 2.4 + LVESD^3^)] (20).

#### Blood pressure and heart rate measurement

**Figure 2:**
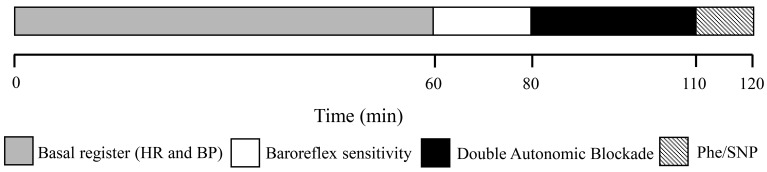
Schematic representation of the cardiovascular autonomic experimental procedures. BP: blood pressure; HR: heart rate; Phe: phenylephrine; SNP: sodium nitroprusside.

Forty-eight hours after the echocardiographic examination, all animals were anesthetized with 80 mg/kg ketamine and 10 mg/kg xylazine, and a polyethylene catheter (PE-50 soldered to PE-10, Intramedic; Clay Adams, USA) was implanted into the left femoral artery and vein. The catheters were tunneled subcutaneously and exteriorized in the nape. To prevent blood from clotting, the catheters were filled with heparinized saline solution 500 IU/mL. Subsequently, the rats were allowed to recover for 24 h prior to the cardiac sympathovagal assessment protocol, which was performed without anesthesia. The arterial pulse (AP) pressure was measured in conscious rats kept in a quiet environment. The mean blood pressure (MBP) was recorded using a pressure transducer (MLT0380, ADInstruments, Australia) (Figure 2). Additionally, the amplified signal (ML110, ADInstruments) was fed to a computer acquisition system (LabChart 7 Pro, ADInstruments). MBP and heart rate (HR) were calculated from the arterial pulse pressure.

#### Cardiac sympathovagal balance

The influence of sympathetic and parasympathetic autonomic tone on HR was assessed by the administration of propranolol (5 mg/kg *iv*, Sigma-Aldrich, USA) and methylatropine (4 mg/kg *iv*, Sigma-Aldrich), respectively. The femoral artery catheter was attached to a pressure transducer (MLT844, ADInstruments), which converts blood pressure fluctuations to electrical signals. Next, the signals were amplified using a bridge amplifier (FE117, AD Instruments), and the pulsatile AP was continuously sampled (2 kHz) using an IBM/PC computer equipped with an analog-to-digital interface (ML866, ADInstruments). After 30 min of basal HR recording, methylatropine was injected in half of the rats in each group, and the HR was recorded for the following 15 min to assess the vagal blockade effect upon it. Furthermore, propranolol was injected into the same rats, and the HR was recorded for another 15 min to determine the intrinsic HR (iHR). For the other half of the rats, the methylatropine-propranolol sequence was reversed to assess the sympathetic blockade effect on the HR following the same recording procedure (15 min each) for each drug to determine the iHR. The data from the methylatropine-propranolol and propranolol-methylatropine sequences were pooled to provide the basal HR (prior to any drugs) and the iHR. After the double pharmacological blockade, doses of 32.0 μg/kg sodium nitroprusside and 16.0 μg/kg phenylephrine were administered for the evaluation of the autonomic blockade (Figure 2).

#### Analysis of HR variability and blood pressure variability

HR and systolic BP variability analyses were performed using custom computer software (CardioSeries v2.0, http://sites.google.com/site/cardioseries) (17). The software was designed to perform a time-frequency analysis of cardiovascular variability, allowing for the precise adjustment of the parameters related to a frequency domain analysis (e.g., interpolation rate, segment length, and frequency band boundaries). As the software did not perform data sampling, beat-by-beat time series were generated and loaded into CardioSeries software. The baseline arterial pressure and pulse interval series obtained from 60-min recordings were processed by computer software (LabChart v7.0, ADInstruments) that applied an algorithm to detect cycle-to-cycle inflection points in the pulsatile arterial pressure signal, thus determining beat-by-beat values of systolic blood pressure (SBP). Beat-by-beat pulse interval series were generated from pulsatile arterial pressure signals by measurement of the time interval between adjacent systolic peaks. Subsequently, the beat-by-beat series pulse interval and SBP series were converted into data points every 100 ms using cubic spline interpolation (10 Hz). The interpolated series were divided into half-overlapping sequential sets of 512 data points (51.2 s), which were tested for the stationary characteristic. It should be noted that cardiovascular variability analysis requires at least a weakly stationary data series (i.e., mean and covariance stability over time) (22).

In the current study, an experienced researcher visually inspected the segments of an interpolated time series (i.e., pulse interval or SBP value) searching for transients that could affect the calculation of power spectral density (PSD). To confirm that the visual inspection was performed properly, a Hanning window was used to attenuate the side effects. Additionally, the spectra of all segments were calculated using a direct fast Fourier transform algorithm for discrete time series. All segments were visually inspected for abnormal spectra. Finally, considering the results of the time series and spectra inspections, nonstationary data were not considered for the PSD calculation. The spectra were integrated in low-frequency (LF; 0.2-0.75 Hz) and high-frequency (HF; >0.75-3 Hz) bands, and the results are reported as absolute (ms^2^ or mmHg^2^) and normalized units. The normalized values were derived by calculating the percentage of LF and HF power with respect to the total power of the spectrum minus the very low-frequency band (VLF; <0.2 Hz) power (23). The band that comprises the LF oscillations of HR [R-R interval (RRi)] represents both sympathetic and vagal modulation, whereas for blood pressure, it represents only sympathetic modulation. In turn, the HF oscillation of HR (RRi) corresponds only to the vagal modulation of the heart (22).

#### Baroreflex sensitivity (BRS) - pharmacological test

Baroreflex sensitivity was determined using the method described by Head and McCarty (24). The changes in MBP were elicited by alternating bolus injections of phenylephrine (0.1 to 16.0 μg/kg) and sodium nitroprusside (0.1 to 32.0 μg/kg). The MBP and HR were measured before and immediately after the injection of phenylephrine (or sodium nitroprusside) when arterial pressure achieved a new steady-state level. The two parameters were then allowed to return to baseline, after which the next injection was administered. A total of at least six increases and six decreases in MBP of different degrees were elicited in each rat. Baroreflex sensitivity was quantified by the slope of the regression line obtained by the best-fit points showing changes in HR and BP in relation to the baseline values (25).

### Statistical analysis

The data are reported as means±SE. A two-way ANOVA was performed to evaluate the effects of ovariectomy and aerobic physical training in all the study groups. When appropriate, *post hoc* comparisons were performed using the Student-Newman-Keuls test. For comparison between two groups, the Student's *t*-test for independent measures or the Mann-Whitney Rank Sum test was used as required. Differences were considered significant at P<0.05. All statistical tests were performed using SigmaPlot software (version 11.0; Systat Software Inc., USA).

## Results

Supplementary Table S1 shows the anthropometric and hemodynamic parameters of all study groups. The ovariectomized SHRs had higher final weights and lower left ventricular relative weights than SHR sham animals. Physical training in ovariectomized SHRs attenuated the reduction in the left ventricular relative weight. Aerobic physical training reduced all the BP parameters.

Supplementary Table S2 shows the values of the cardiac morphology and functionality analysis. Ovariectomy reduced PWT, IVST, LVEDD, and LVESD. In turn, physical training associated with ovariectomy further reduced the PWT. The cardiac functionality showed that anesthetized ovariectomized rats had an increase in LVESV. Physical training did not promote significant changes in cardiac functionality.

**Figure 3:**
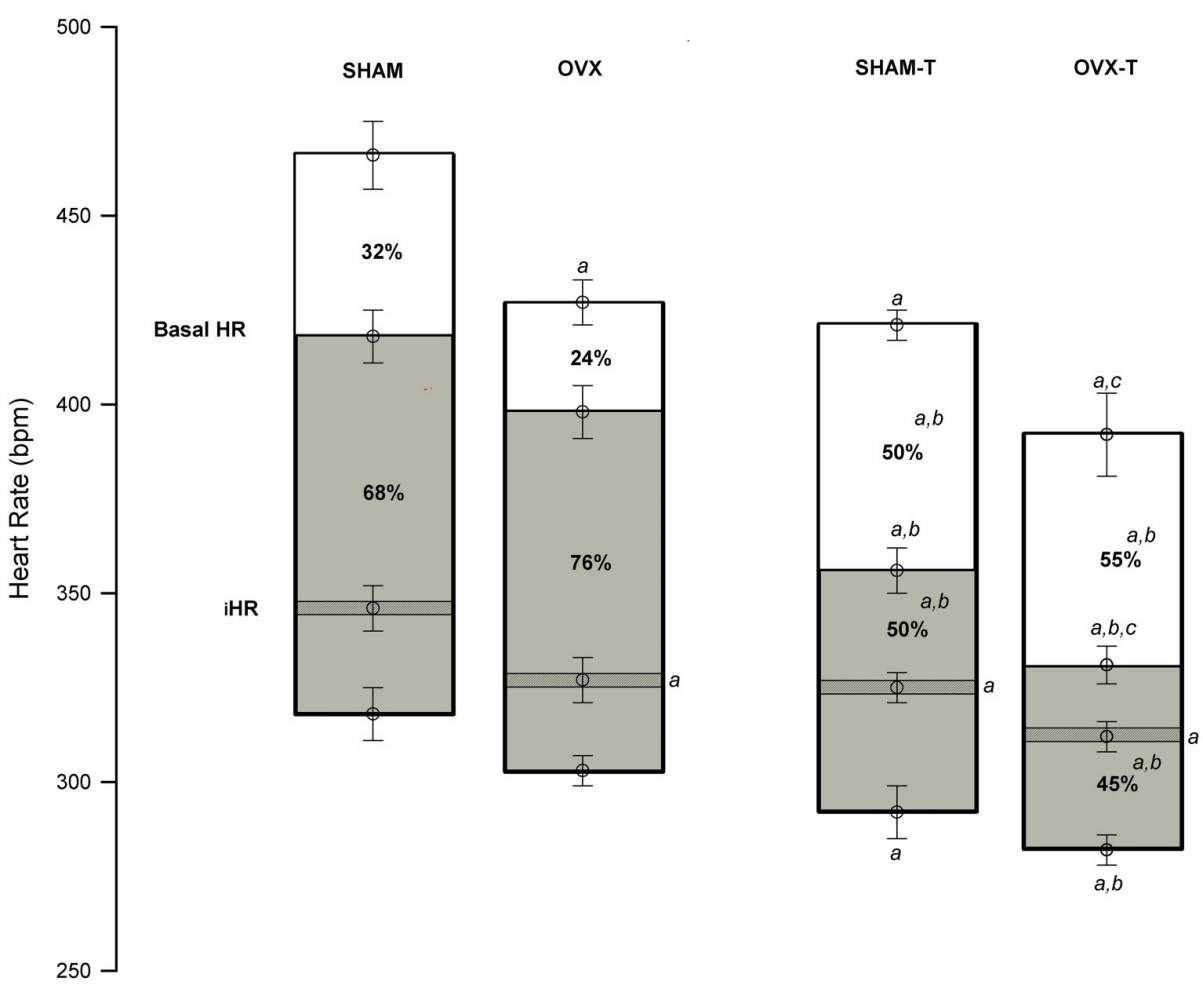
The bar graphs show the delta (Δ) of heart rate (HR) after methylatropine (white box) and propranolol (gray box) injection in the SHAM and ovariectomized (OVX) groups of sedentary (SHAM and OVX) and trained (SHAM-T and OVX-T) animals. All data are reported as means±SE. ^a^P<0.05 *vs* SHAM; ^b^P<0.05 *vs* OVX; ^c^P<0.05 *vs* SHAM-T (ANOVA). iHR: intrinsic HR.

Supplementary Table S3 presents the cardiac autonomic balance results after the double pharmacological block with methylatropine and propranolol. Ovariectomy and physical training induced lower HR values after methylatropine, whereas only physical training reduced baseline HR. For this reason, the HR variations (ΔHR) from baseline HR were greater after methylatropine in the trained groups than in the untrained groups. After propranolol administration, the trained groups showed lower HR and smaller ΔHR compared to the untrained groups. The double block showed that both ovariectomy and physical training reduced the pacemaker iHR. Figure 3 illustrates the balance of cardiac autonomic tone after a double pharmacological block and shows the HR delta (Δ) of response in which physical training reduced sympathetic autonomic tone and/or increased vagal autonomic tone.

The cardiovascular autonomic modulation and BRS results are shown in Supplementary Table S4. Ovariectomy and physical training reduced the variance and LF oscillations of the BPV. As for the BRS assessment, no intergroup differences were observed.

## Discussion

The deprivation of ovarian hormones in SHRs promoted morphological and functional adaptations in the heart; however, it did not interfere with cardiac autonomic parameters. Aerobic physical training reduced HR and BP, promoted morphological adaptations, and altered the balance of cardiac autonomic tone, favoring the vagal component even in ovariectomized animals.

Physical training attenuated blood pressure values in ovariectomized and sham animals. This seems to be due to many factors, including reduced peripheral vascular resistance and improved endothelial function (26,27), reduced oxidative stress (28), and decreased cardiac vascular sympathetic activity (29). Aerobic exercise reportedly reduces BP in hypertensive patients by restoring the balance between excitatory and inhibitory neurotransmitters and between pro- and anti-inflammatory cytokines in the paraventricular nucleus (30).

Physical training for 12 weeks also attenuated the baseline and intrinsic HR of awake animals (Supplementary Table S3). However, the HR obtained during the echocardiography examination did not show the same values (Supplementary Table S2). The difference in HR values was caused by anesthesia (isoflurane) used during the exam. In fact, the reduction in HR observed in the awake animals, as well as the reduction in BP, suggests the efficiency of the physical training applied to these animals. This efficiency was also noted in the changes observed in the cardiac tonic autonomic control. Among the adaptations of the cardiovascular system that we can expect from physical training, autonomic balance regulation seems evident (29,31,32). This was observed in the double pharmacological block protocol. In this case, we observed greater variations in HR in untrained animals after propranolol than methylatropine administration, indicating a predominance of sympathetic autonomic components in determining baseline HR. However, the HR response to propranolol administration was attenuated in animals subjected to aerobic physical training, whereas the HR response to methylatropine administration was enhanced. Thus, these results confirmed that physical training can influence cardiac tonic autonomic control by promoting a rearrangement in cardiac sympathovagal balance characterized by reduced sympathetic influence of the autonomic component and/or an increase in the vagal influence of the autonomic component in determining the baseline HR, regardless of ovarian hormone deprivation.

On the other hand, neither ovarian hormone deprivation nor aerobic physical training influenced HRV parameters in SHRs. The effects of ovarian hormone deprivation on HRV in experimental models and in women remain inconclusive. In the clinical context, after menopause, a deficit in cardiac autonomic regulation has been observed and attributed to HRV reduction (3,11). Likewise, lesser sympathetic and greater vagal modulation have been demonstrated in premenopausal *vs* postmenopausal women (3,33). In our study, ovariectomy in SHRs did not impair cardiac tone or autonomic modulation. However, other studies have suggested a direct relationship between ovarian hormone deprivation and the lower bioavailability of nitric oxide (NO), which affects autonomic HRV modulation, sympathovagal tonic balance, and BRS (12,34). In contrast, HRV and BRS did not differ between the normotensive sham and ovariectomized rats regardless of aerobic physical training (32,34). Furthermore, the effects of early menopause (at 10 weeks of life) *vs* physiological menopause both in normotensive rats at 82 weeks of age showed similar hemodynamic and autonomic parameters, suggesting that the differences in the evaluated variables would be more closely related to aging than ovarian hormone deprivation (17).

A study did not identify changes in LF and HF oscillations in SHR rats subjected to ovariectomy. However, hormone replacement therapy consisting of β-estradiol altered the sympathovagal balance characterized by an increase in the LF power bands and a reduction in the HF bands that increased the LF/HF ratio (35). Another finding is that ovariectomy seems to reduce the BPV values, and the association with physical training potentiated this reduction. This is notable because the opposite effect was observed in ovariectomized Wistar rats (17). The cause of BPV reduction in SHRs after ovariectomy is uncertain and requires further investigation. No changes were observed in HRV or BRS in the trained animals. Other authors reported negative effects of ovariectomy, but positive effects of physical training (18,29,34). However, in the current study, ovariectomy did not influence these autonomic parameters, which might explain our finding that physical training did not improve HRV and BRS. However, further studies are required to clarify these differences.

Evaluations of cardiac functionality and morphology revealed that SHRs show significant changes in these parameters, mainly between the second and third months of life (36,37). In such cases, increases in left ventricle mass, cardiac fibrosis, and changes in other morphological parameters induce cardiac functionality impairments that may progress to heart failure. Studies of ovariectomized SHRs showed important pathological concentric hypertrophy, increased interstitial myocardial fibrosis, and cardiac cell apoptosis at 23-40 weeks of life (38). Notably, in our study, ovariectomy did not negatively impact cardiac morphology and functionality. In addition, the interaction with the physical exercise factor promoted attenuation of PWT.

The causes of these observations are not yet established and suggest that these morphological and functional adaptations might be associated with the regulation of cardiac autonomic tone balance that favors the vagal autonomic component. Other possible explanations might be related to the reduction in peripheral resistance resulting from the improvement of endothelial function and inflammatory profile and attenuation of the renin-angiotensin-aldosterone system as suggested by some authors (8,39,40). However, it is important to note the effects of physical training on cardiac function. An intragroup analysis of the SHAM groups showed that physical training increased ejection volume and shortened ejection fraction; however, these changes were not observed in the intragroup comparison of the OVX groups. The causes for this are uncertain, although in some parameters, the ovariectomized animals had higher baseline values.

In conclusion, aerobic physical training in SHRs with preserved ovarian function reduced BP and sympathetic tone and increased vagal tonic influence but did not affect cardiac function. Ovarian hormone deprivation in SHRs increased BP and promoted important morphofunctional adaptations. Aerobic physical training in ovariectomized SHRs appears to contribute to positive morphological and functional adaptations. However, the mechanisms involved in this process remain uncertain and require further investigation.

## Supplementary Material

Click to view [pdf].
